# Outcomes of Femoral Neck System in Treating Intra-capsular Neck of Femur Fractures in a Young Adult: A Case Report

**DOI:** 10.7759/cureus.66661

**Published:** 2024-08-11

**Authors:** Swaroop Solunke, Mohammed Talha Muneer, Ashwin Deshmukh, Ishan Shevate, Abhishek Nair

**Affiliations:** 1 Orthopaedics, Dr. D. Y. Patil Medical College, Hospital and Research Centre, Dr. D. Y. Patil Vidyapeeth, Pune (Deemed to Be University), Pune, IND

**Keywords:** intra-capsular neck of femur (icnf), femoral neck system (fns), avascular necrosis (avn), dynamic hip screw (dhs), cancellous screw (cs)

## Abstract

The introduction of the Femoral Neck System (FNS) represents a promising alternative to traditional cancellous cannulated (CC) screw fixation for managing intra-capsular neck of femur (ICNF) fractures. This case report aims to validate its safety and report the outcomes in a young patient. The findings demonstrate that the FNS possesses excellent biomechanical properties and provides significantly greater overall construct stability bearing in mind, that it was used in a Pauwels Classification Grade 3 ICNF fracture.

## Introduction

Intra-capsular neck of femur (ICNF) fractures are frequent and account for more than half of cases of all hip fractures [1]. Orthopedic surgeons face a great deal of difficulty in managing these injuries, which are common in the elderly. ICNF fractures in adults under 50, however, are rare and frequently the consequence of high-energy trauma. To encourage endosteal healing, anatomic reduction is thought to be crucial in the treatment of these fractures [2]. In these fractures, the periosteum's cambium layer, which normally prevents callus formation, is absent [2]. In comparison to existing implants, the newly created Femoral Neck System (FNS) offers potential biomechanical advantages for treating ICNF fractures. The objective of our study was to highlight the use of FNS for ICNF fracture in a young patient.

## Case presentation

**Figure 1:**
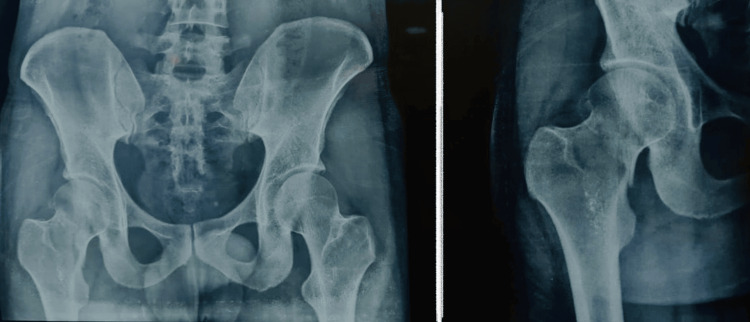
(Left panel) Pre-operative X-ray of the pelvis and both hips in an anteroposterior view. (Right panel) Close-up anteroposterior view of the right hip.

Our patient, a 34-year-old male, came following a high-energy trauma history with complaints of pain in the right groin. Clinically the patient presented with a flexed, externally rotated, and shortened right limb with no open wounds. Neurovascular examination was non-contributory. Radiographic evaluation done using an X-ray of the pelvis and both hips anteroposterior view as seen in Figure 1 revealed a Pauwels Classification Grade 3 ICNF fracture on the right side.

The minimally invasive FNS was chosen over a sliding hip screw with a de-rotation screw for this patient due to its superior compression and angular stability. Moreover, there is less chance of mal-reduction with the FNS implant insertion than with a sliding hip screw device because the bolt does not need to be "screwed in."

**Figure 2:**
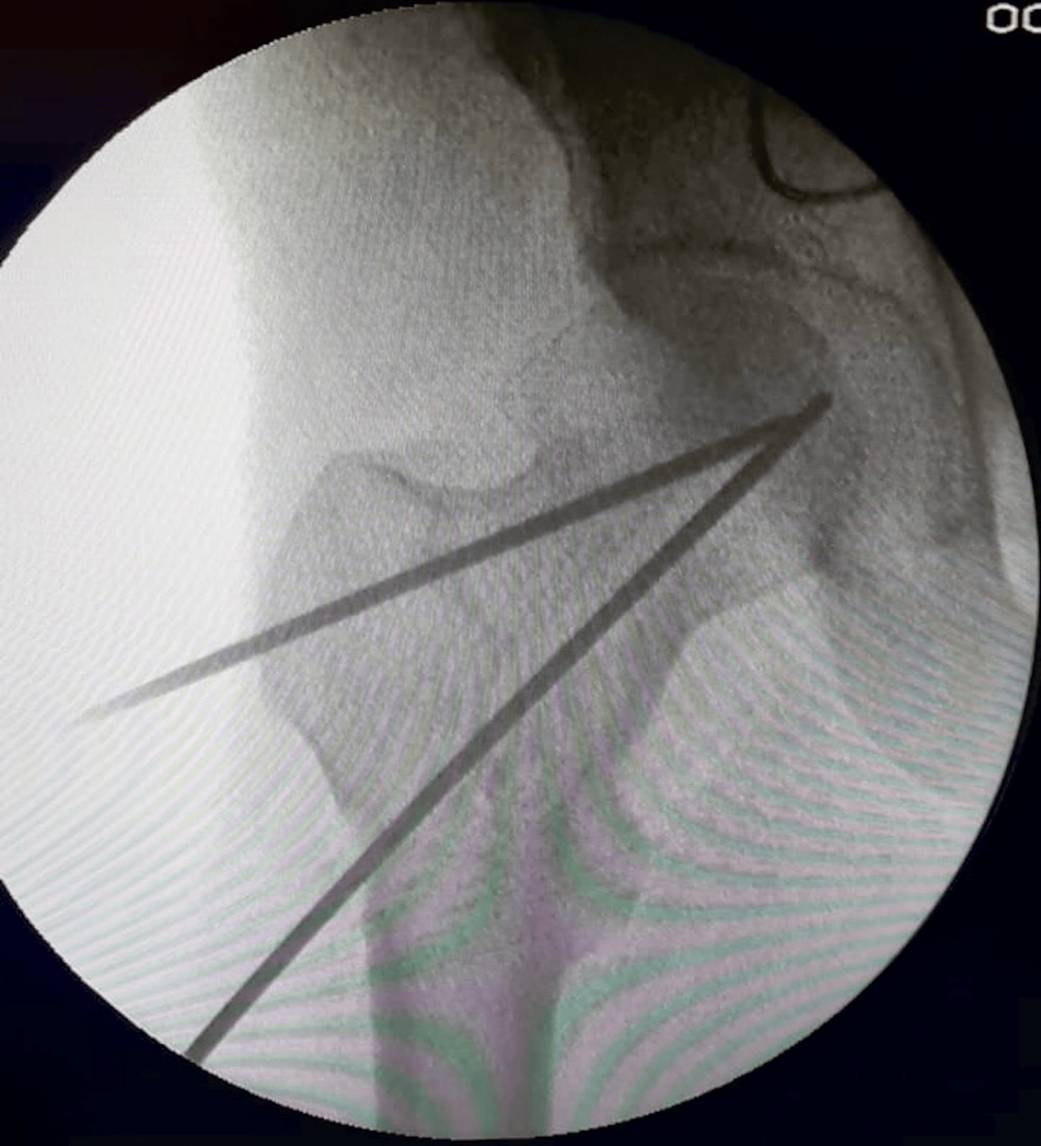
C-arm image showing provisional fixation of the fracture intraoperatively using K-wires.

The lateral portion of the femur, immediately distal to the vastus ridge, was incised to an extent of 5 to 10 centimeters. The femoral head was measured, and the implant size was established following the insertion of guide wires. The reduction was able to be maintained during insertion without the femoral head moving incorrectly as the bolt was smooth as shown in Figure 2.

**Figure 3:**
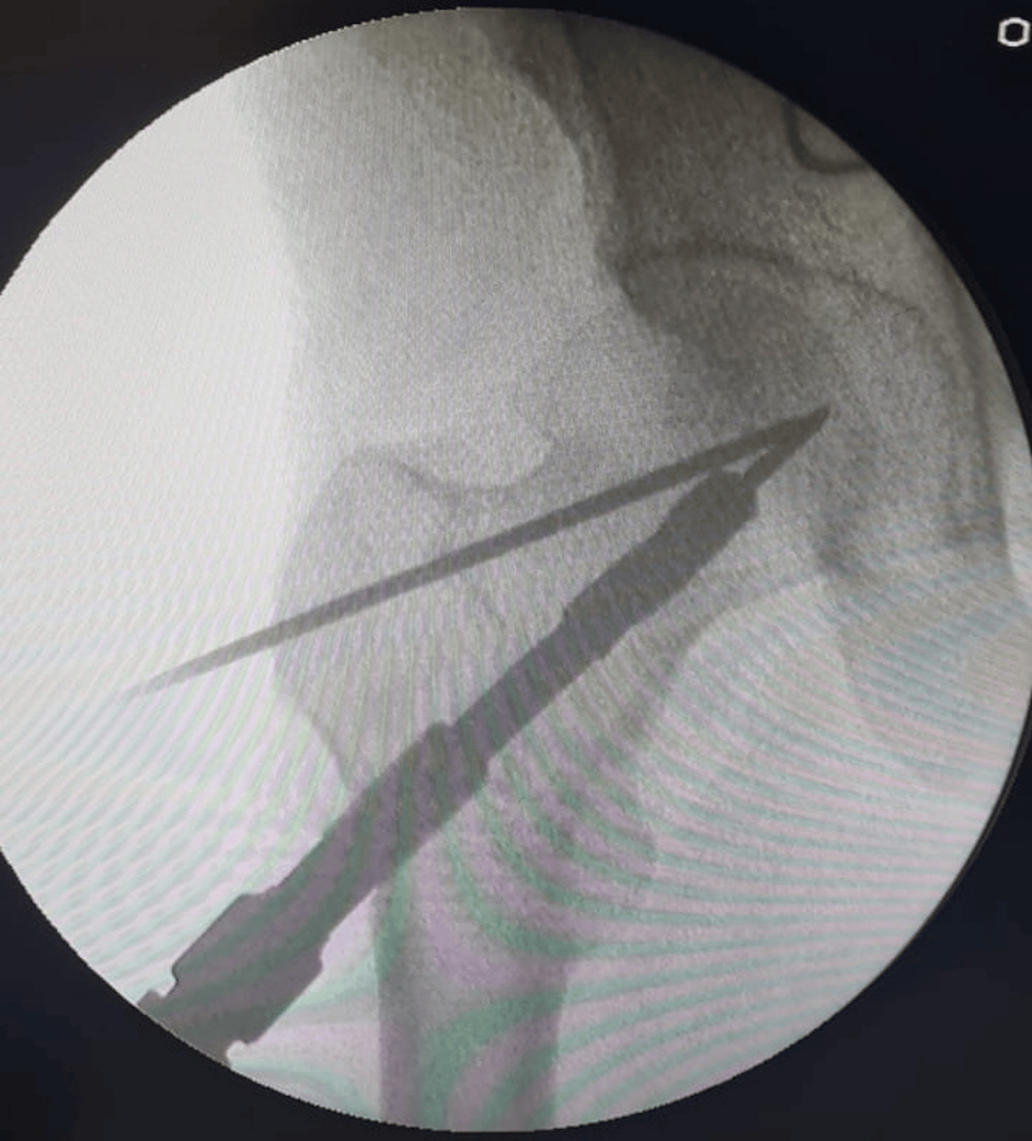
Confirmation of implant position intraoperatively using an image intensifier.

To establish a single permanent assembly, the anti-rotation screw was put through the bolt. The plate location was fixed after the anti-rotation screw was put in. Before drilling and screwing in the anti-rotation screw, the plate's position was verified with the image intensifier to make sure it was centered concerning the femur shaft as shown in Figure 3.

**Figure 4:**
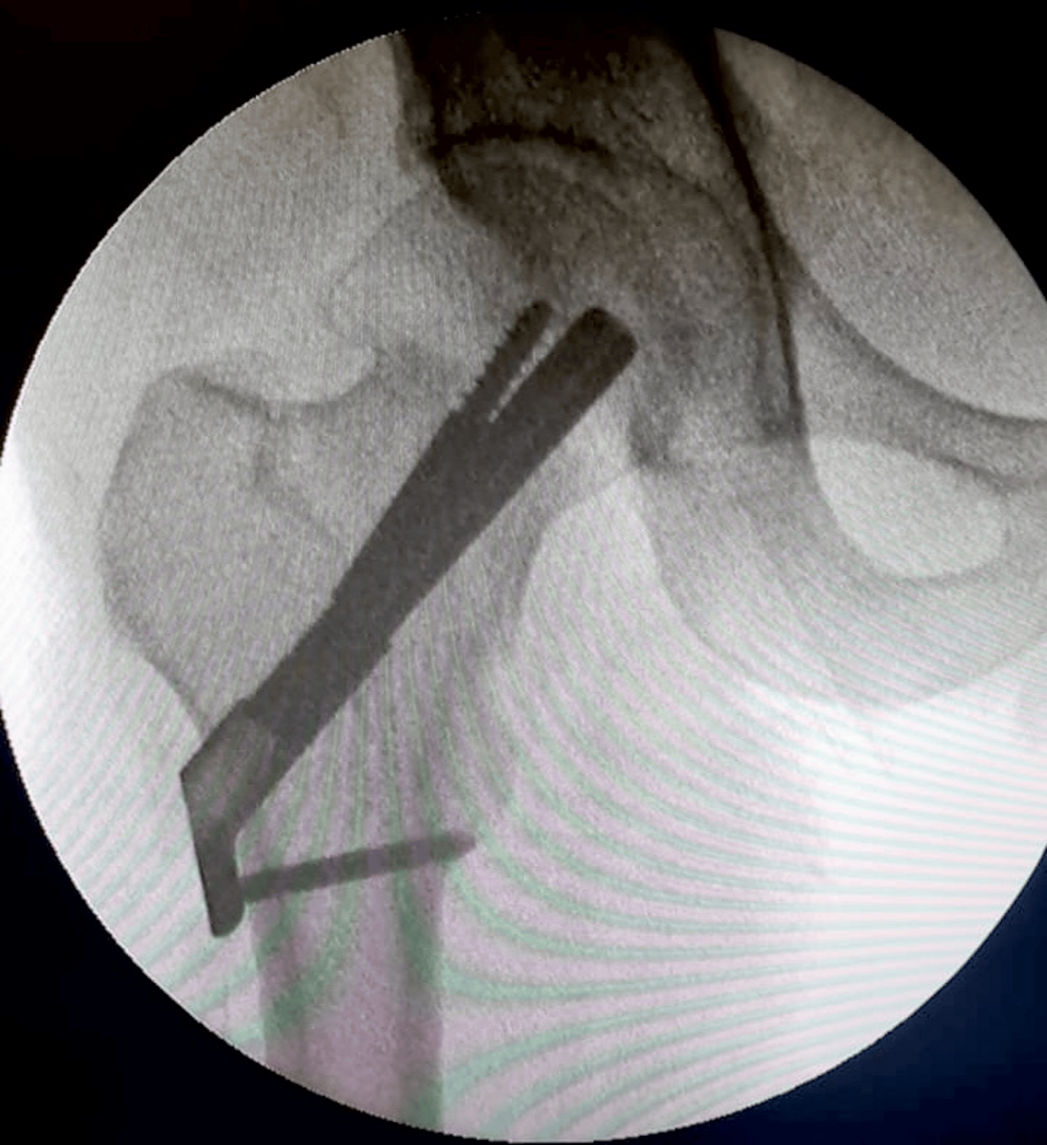
Final intraoperative C-arm image with the Femoral Neck System in situ.

A targeting jig allowed for the simple insertion of all the FNS implant’s components using a small number of instruments as seen in Figure 4.

**Figure 5:**
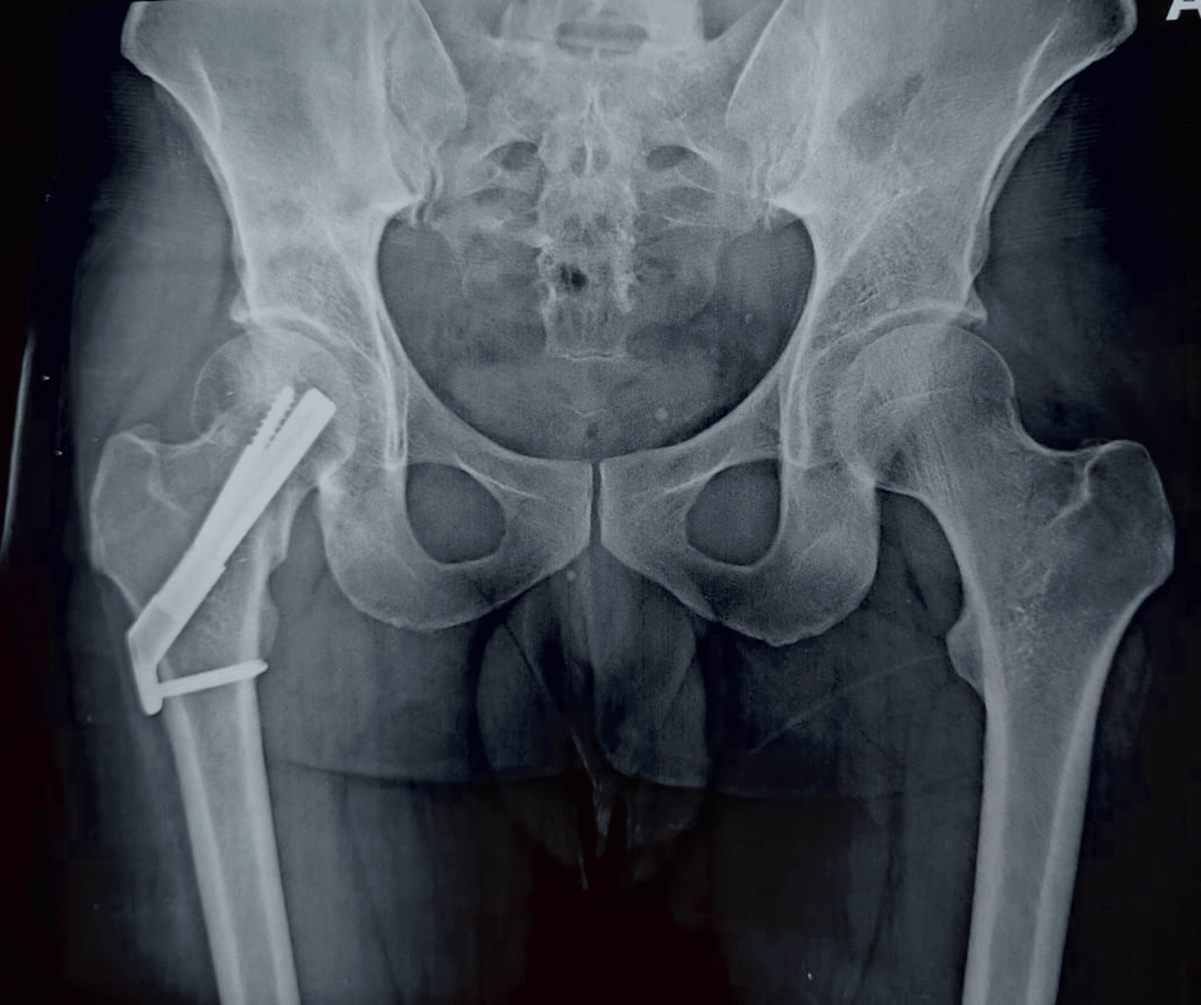
Immediate postoperative X-ray of the pelvis and both hips in an anteroposterior view showing the Femoral Neck System in situ.

For this patient, a two-hole plate was chosen due to the fracture's vertical morphology and inferior comminution as shown in Figure 5.

**Figure 6:**
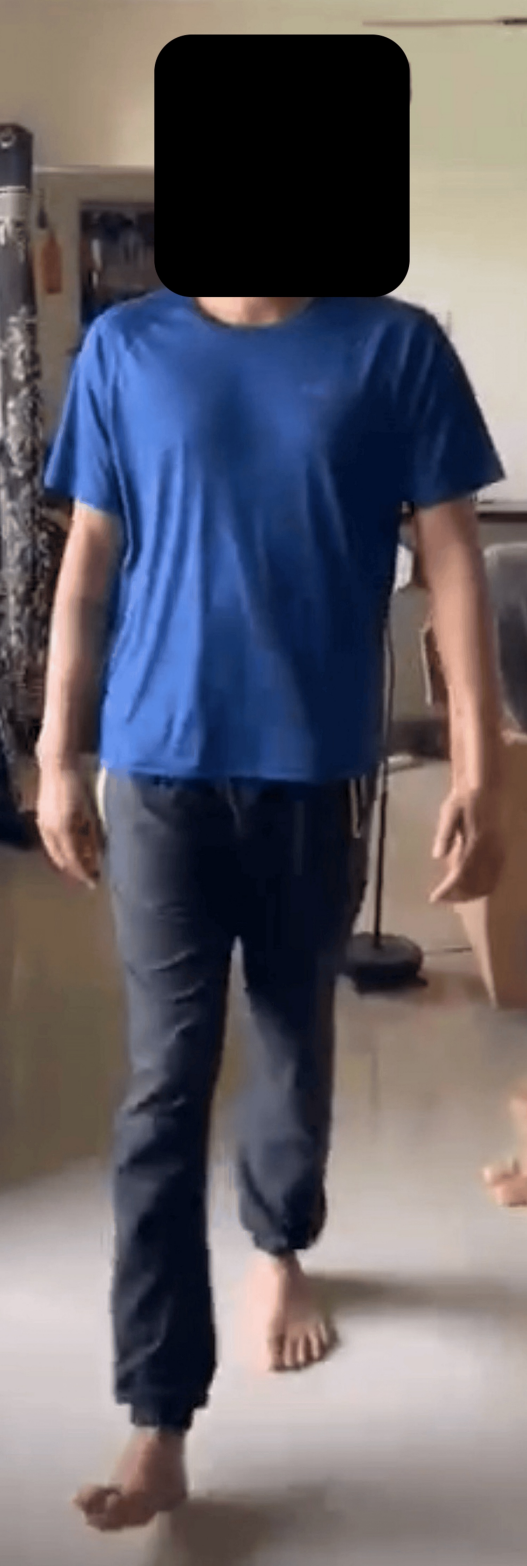
Six months post-surgery follow-up showing the patient walking without a limp.

**Figure 7:**
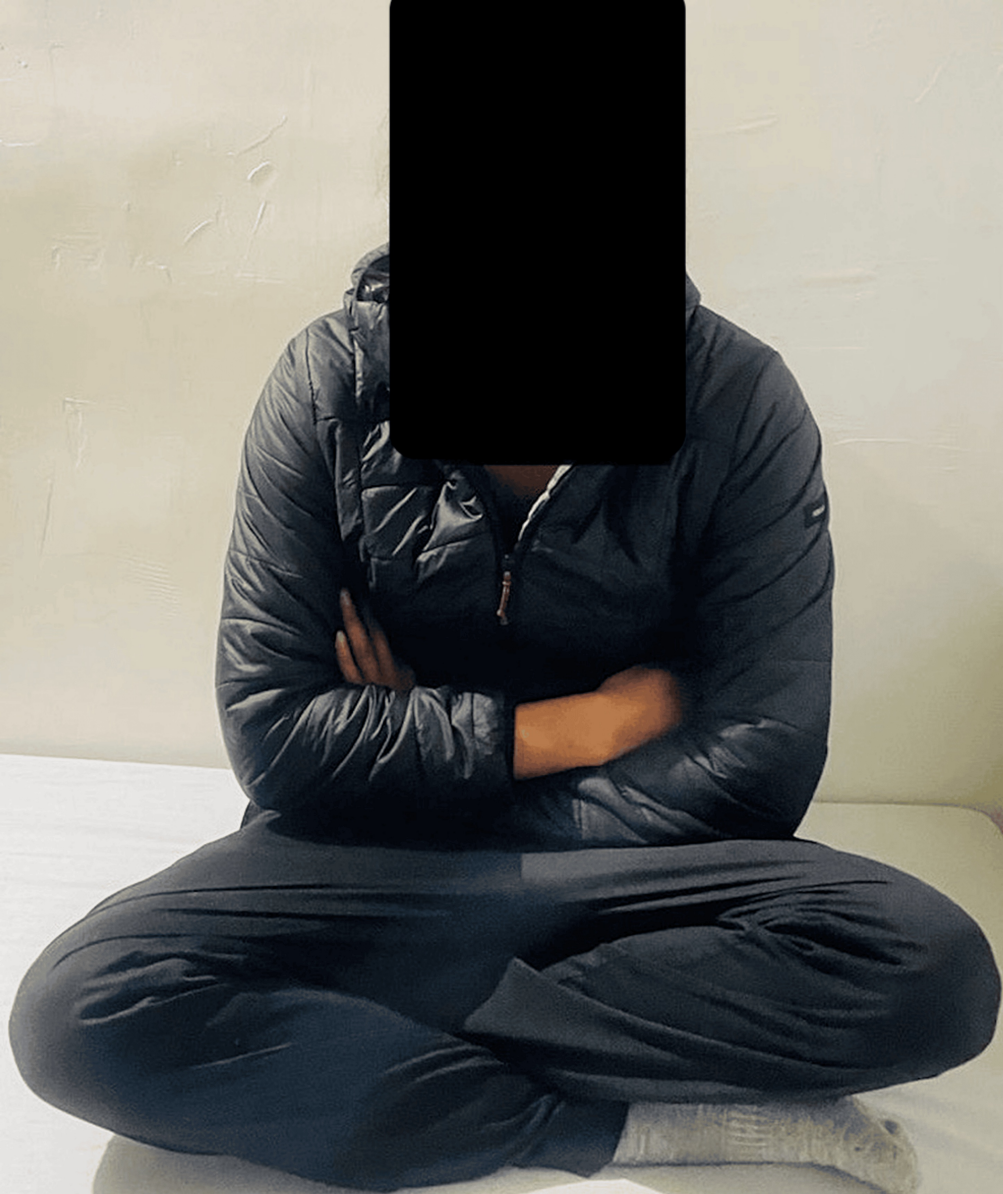
Six months post-surgery follow-up showing the patient exhibiting a full range of motion while sitting cross-legged.

The patient was discharged and encouraged to weight bear. The patient was regularly followed up and at six months post-surgery, he had achieved full range of motion in the right lower limb. The ICNF fracture had healed, the FNS implant remained intact, and the patient was walking pain-free without a limp as seen in Figures 6-7.

**Figure 8:**
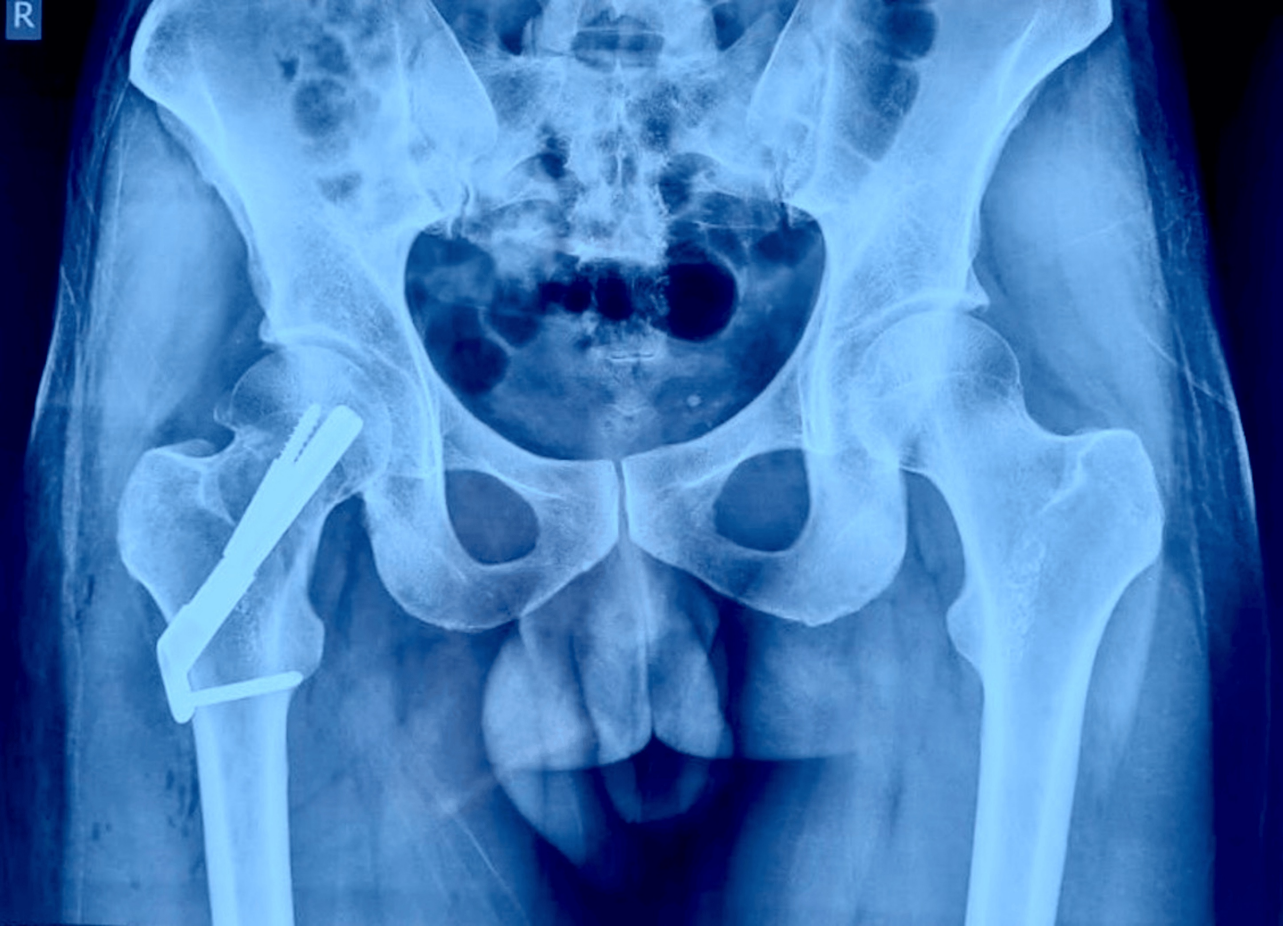
X-ray of the pelvis and both hips in an anteroposterior view taken at the one-year follow-up.

Follow-up X-ray of the pelvis and both hips anteroposterior view taken after one year showing a well-placed implant with signs of fracture union implying successful management of ICNF fracture in young adults using FNS as shown in Figure 8.

## Discussion

ICNF fractures in young adults remain a challenging and unresolved issue. Displaced fractures have more complications like non-union and avascular necrosis (AVN) of the femoral head [3,4]. There is the absence of a cambium layer of periosteum that prevents callus formation in these fractures [2]. The ideal management strategy for ICNF is surgical and may involve either internal fixation or arthroplasty, depending on bone quality, fracture severity, and the patient's age [5]. Treatment of a young adult's femoral neck fracture in clinical practice remains difficult due to the significant risk of postoperative sequelae, including non-union, AVN, and neck shortening.

The key elements to minimizing the aforementioned problems are perfect anatomical reduction and firm, stable internal fixation [6]. The best surgical method for fixing femur neck fractures is still up for debate. A few of its attributes should be shorter surgical times, lower blood loss during the procedure, lower rates of postoperative morbidity and mortality, shorter hospital stays, lower costs, and early patient mobilization to enhance the patient's ability to return to work sooner and lessen the risks associated with bed rest, such as deep vein thrombosis, pulmonary complications, and bed sores. DePuy Synthes (Raynham, MA, USA) has recently introduced the FNS, which offers several mechanical benefits for internal fixation by combining compression and anti-rotation properties. The design of the FNS, featuring a screw-plate construct, provides a more robust fixation. Additionally, the inclusion of a blade and an anti-rotation screw enhances both axial and rotational stability. Biomechanical research indicates that the FNS implant surpasses traditional cannulated screws (CS) and the dynamic hip screw (DHS) in terms of axial and rotational stability [7,8]. FNS offers a stable and secure fixation to lessen varus deformity following surgery. Given the instability and the fracture pattern in our patient, the FNS two-hole side plate was ideal to use in our case.

A well-designed follow-up plan was advised to the patient at regular intervals. At the six-month follow-up, the patient had achieved a full range of motion. At the one-year follow-up, the patient was able to walk pain-free and without a limp.

## Conclusions

The use of an FNS implant encouraged early weight bearing and better fracture healing. This study shows that FNS is a safe and superior treatment option for ICNF fractures in young adults.
